# Global trends in dementia care research in the context of COVID-19: bibliometric analysis

**DOI:** 10.3389/fmed.2024.1388767

**Published:** 2024-07-11

**Authors:** Rafael Gómez-Galán, Ángel Denche-Zamorano, Maria Mendoza-Muñoz, Damián Pereira-Payo, Sabina Barrios-Fernández, Laura Muñoz-Bermejo

**Affiliations:** ^1^Research Group on Physical and Health Literacy and Health-Related Quality of Life (PHYQOL), University Centre of Mérida, University of Extremadura, Mérida, Spain; ^2^Promoting a Healthy Society Research Group (PHeSO), Faculty of Sport Sciences, University of Extremadura, Cáceres, Spain; ^3^Research Group on Physical and Health Literacy and Health-Related Quality of Life (PHYQOL), Faculty of Sport Sciences, University of Extremadura, Cáceres, Spain; ^4^Health, Economy, Motricity and Education (HEME) Research Group, Faculty of Sport Sciences, University of Extremadura, Cáceres, Spain; ^5^Spain Social Impact and Innovation in Health (InHEALTH), University Centre of Mérida, University of Extremadura, Mérida, Spain

**Keywords:** SARS-CoV-2, major neurocognitive disorder, caregivers, scientometrics, dementia

## Abstract

Alzheimer’s disease is the most common type of dementia, producing a deterioration in the activities of daily living which can lead to the need for care provision. COVID-19 impacted their quality of life and in this care delivery. This study aimed to analyse most productive and prominent authors, the journals and countries with the highest number of publications, the most cited documents and the most used keywords. Publications were retrieved from journals indexed in the Main Collection of the Web of Science (WoS) and analysed using the traditional laws of bibliometrics. A total of 376 documents were found. The WoS categories with the highest number of publications accumulated were “Geriatric Gerontology” and “Gerontology.” Clarissa Giebel was the most productive (23 papers) and most cited (with 569 citations) co-author. The Journal of Alzheimer’s Disease (21 papers) published the most number of documents. The manuscript “2021 Alzheimer’s Disease Facts and Figures” was the most cited. Four thematic clusters related to mental health, telemedicine, care and well-being were found among the authors’ keywords. Research networks exist worldwide, with the United States of America and England leading the scientific output. These results may be of interest to researchers, publishers and professionals interested in this subject, as they provide current information on publications related to this topic.

## 1 Introduction

Dementia is an acquired syndrome characterised by a set of signs and symptoms that produce a persistent impairment of cognitive functions and affect functional capacity (1). With 60–80% of cases, Alzheimer’s disease (AD) is the most common cause of dementia in the elderly (2), with slow destruction of the cerebral cortex, clinically debuting with a progressive process of cognitive and functional decline (3). Then, increased life expectancy has caused a proportional increase in the risk of dementia (4) as greater age increases the risk of dementia leading to an increased demand for elderly care, especially in western industrialised countries (5). Moreover, there are currently 47 million people estimated with dementia worldwide (6), and this prevalence increases by 5% every five years in people older than 69 years onwards and is higher in women, probably due to their greater longevity (7).

Dementia results in a gradual loss of activities of daily living (ADL) management and the need for caregiver assistance in ADL performance increases. Caring for a person with AD requires great dedication and commitment due to the irreversible and progressive nature of the disease, its long duration and the deterioration in multiple areas of cognitive skills, behavior and personality (8). In addition, caregivers may be required to carry out activities that may not motivate them or for which they feel unprepared (9). The result is a dependent relationship with the caregiver(s), with an increased risk of adverse effects on almost every aspect of their lives, including health, well-being and quality of life, relationships and economic security (10–12). Care for patients with dementia is mainly provided by family caregivers (13, 14), and most of them are women, with this figure accounting for 76% in Spain (15).

At the end of 2019, the first cases of pneumonia of unknown origin (16), which were later named Coronavirus Disease or COVID-19 by the World Health Organisation (WHO) (17) were diagnosed in China. Social distancing and confinement measures increased psychological and behavioral symptomatology in around 60% of people with dementia, which led to approximately 2/3 of their caregivers experiencing stress-related symptoms (18). This situation resulted in stressful experiences when family caregivers were overburdened by the activities of attending to the care needs of their relatives, in a situation already atypical due to the pandemic (19).

Bibliometric studies are statistical methods that quantitatively explore scientific production in an area of interest, allowing for the analysis of general trends in publications, researchers and institutions and their relationships, journals and keywords (20, 21). The main objective of this study was to examine global trends in dementia care research during COVID-19, given that the pandemic is expected to have a major impact on this population and their care. Secondary objectives included identifying the annual publications, the thematic categories that have accumulated the most papers on this object of study, the prolific and prominent authors, the most relevant papers on the subject, the journals that published the most papers and the keywords most used by the authors.

## 2 Method

### 2.1 Design and data source

A descriptive bibliometric analysis was conducted to map scientific research published in journals indexed in the Web of Science (WoS) of Clarivate Analytics on care in people with dementia in the context of COVID-19. The WoS is one of the most widely used sources for bibliometric analysis by researchers, given the large number of indexed journals and the comprehensive information it provides on publications, co-authors, titles, abstracts, keywords, sources, publishers, citations, etc.; and it is frequently used for bibliometric studies on different topics in journals from different publishers (22–25). Then, a search was performed on 6 November 2023 in the WoS Core Collection database to obtain the set of documents with the following search vector: ((TI = (“dementia” OR “alzhe*”) OR AB = (“dementia” OR “alzhe*”) OR AK = (“dementia” OR “alzhe*”)) AND (TI = (“Novel coronavirus 2019” OR “Coronavirus disease 2019” OR “COVID 19” OR “2019-nCOV” OR “SARS-CoV-2” OR “coronavirus-2”) OR AB = (“Novel coronavirus 2019” OR “Coronavirus disease 2019” OR “COVID 19” OR “2019-nCOV” OR “SARS-CoV-2” OR “coronavirus-2”) OR AK = (“Novel coronavirus 2019” OR “Coronavirus disease 2019” OR “COVID 19” OR “2019-nCOV” OR “SARS-CoV-2” OR “coronavirus-2”)) AND (TI = (“Nurs*”) OR AB = (“NURS*”) OR AK = (“NURS*”) OR TI = (“caregiv*”) OR AB = (“caregiv*”) OR AK = (“caregiv*”) OR TI = (“carer*”) OR AB = (“carer*”) OR AK = (“carer*”))) limiting to editions: Science Citation Index Expanded (SCI-Expanded), Social Sciences Citation Index (SSCI) y Emerging Sources Citation Index (ESCI); and as document type, to articles and article reviews. The WoS was searched using the tags TI (Title Search), AB (Abstract Search) and AK (Author Keywords Search). Data were extracted from WoS in .xslx and plain text format and then analysed in Microsoft Excel for Microsoft 365 MSO version 2,206 and with the VoSViewer software version 1.6.18. The criteria for inclusion were: (1) to meet the search criteria; (2) to be scientific articles or reviews; (3) to be related to the topics of the study.

### 2.2 Data analysis

The trend of annual publications was evaluated, obtaining the data from the WoS “Analysis of Results” tool. In the same way, the thematic categories in which the documents were distributed in WoS were obtained. The concentration of publications in authors or Lotka’s Law was used, recognising that in any field of knowledge, the majority of articles come from a small proportion of prolific authors who, once identified, can be studied in isolation (26). This law states that the number of authors making *n* contributions is about 1/*n^a^* of those making one contribution, where *^a^* is often nearly REF (27). This was verified using the coefficient of determination (*R*^2^) calculated in Microsoft Excel. The most cited papers emerged by applying the Hirsch index (*h*-index) to the papers (“*h*” articles cited at least “*h*” or more times), considering these as the most prominent articles. Prominent authors were found by crossing the prolific authors with the most cited papers, considering prolific authors with papers among the most cited papers as prominent authors (28). The concentration of publications in journals or Bradford’s Law was performed by distributing the journals in terciles according to the number of documents published in each journal, establishing as the core of journals in the subject area those that comprised at least 33% of the total number of publications. Thus, the journals are sorted into three groups from the highest to the lowest number of publications: Core (few journals with many publications), Zone 1 (with more journals) and Zone 2 (the bulk of journals, with at least one publication in the topic) (29, 30). Keyword concentration, or Zipf’s Law, which states that “the number of appearances of the *n*th keyword is inversely proportional to *n*,” highlighted the most commonly used keywords in the set of articles, through a discrete count of the number of documents in which each keyword appeared using Microsoft Excel (31). VOSviewer software was used to process and visualise the dataset, performing co-occurrence analysis of author and country co-authorship and word co-occurrence (32, 33).

## 3 Results

### 3.1 Total documents

**Figure 1:**
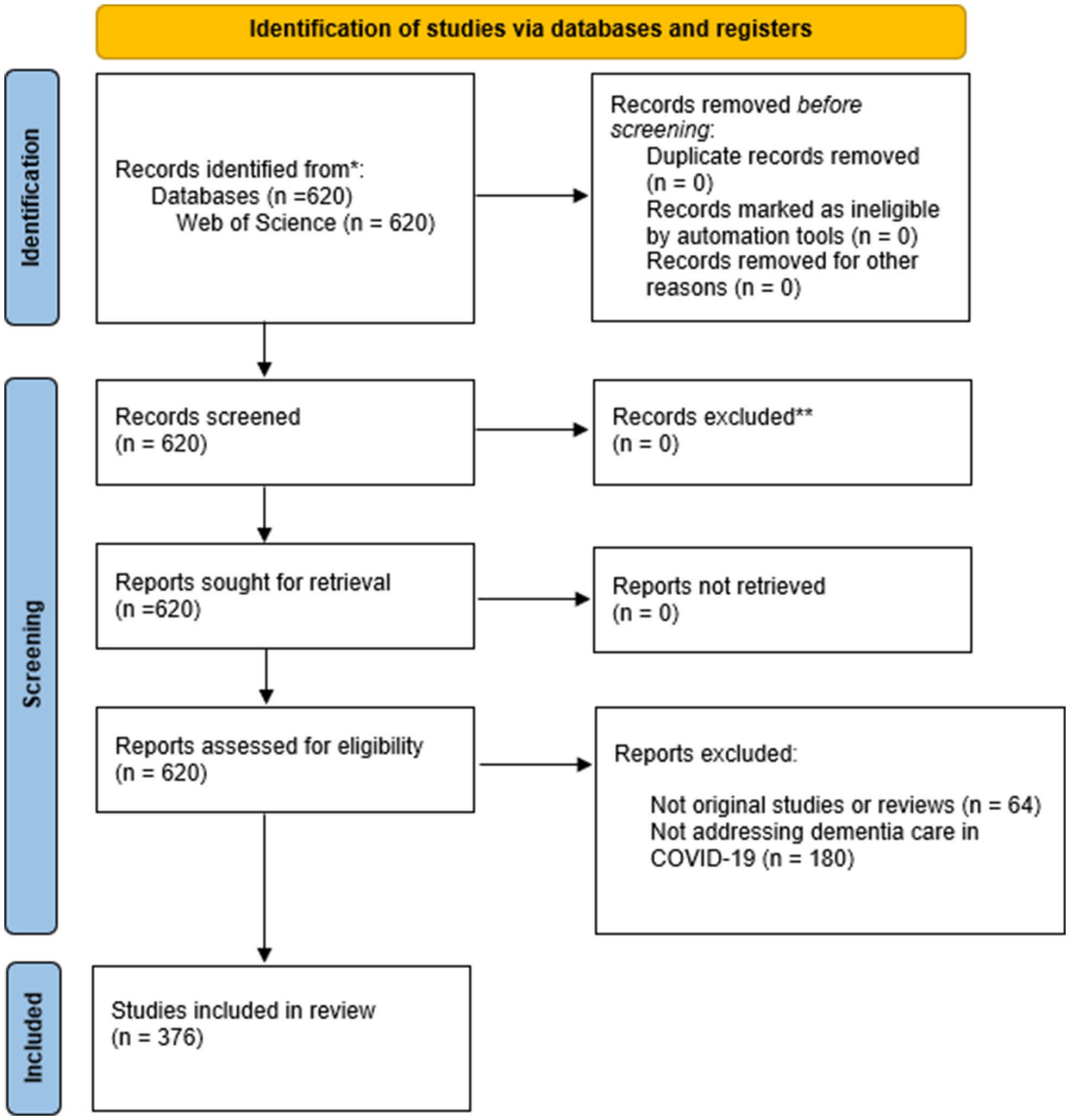
Flow diagram.

A total of 620 documents were obtained. Then, 64 were eliminated as they were not scientific articles or reviews. From the remaining 556, 180 were excluded as they did not deal with dementia care in COVID-19, leaving a total of 376 documents for analysis (Figure 1).

### 3.2 Publication periods and subject categories

**Figure 2:**
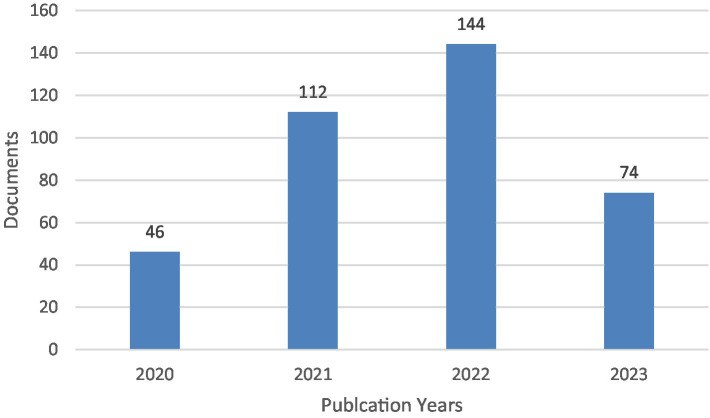
Number of documents by publication year.

A total of 376 articles were published in the period between 2020 and 2023 (Figure 2) (Supplementary Table S1). There was an increase from 2020 to 2022 in the number of publications. In 2023 there was a decrease, but it should be noted that the search was conducted in November 2023.

The 376 papers were distributed across 48 WoS categories. The categories with the highest number of papers were “Geriatrics Gerontology” (129 papers, 34.3%) and “Gerontology” (121 papers, 32.2%), with up to 65% of the total publications related to these thematic categories.

### 3.3 Authors

**Figure 3:**
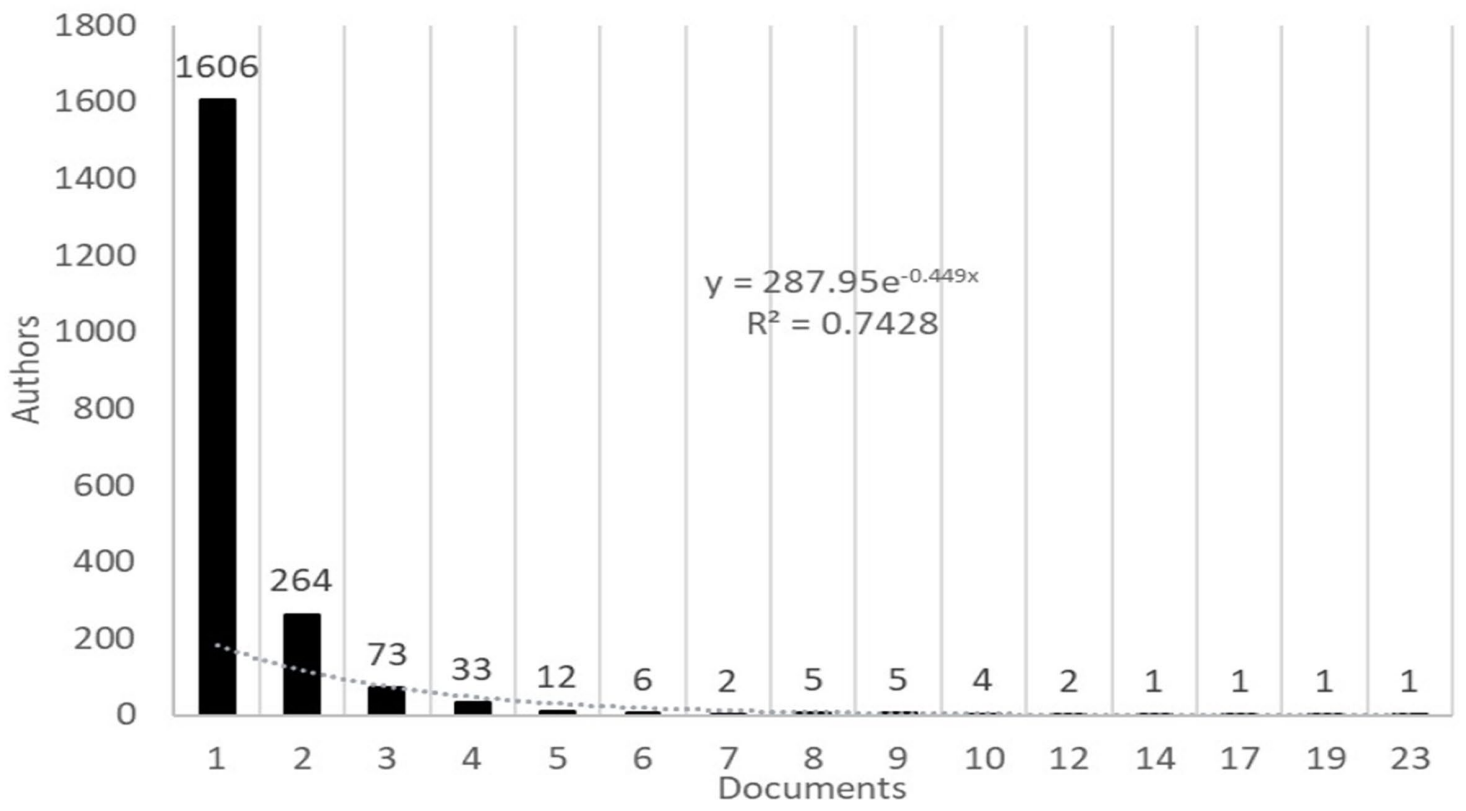
Number of papers published by authors.

Applying Lotka’s Law to the 376 papers that resulted in the scientific output of 2016 authors, it was estimated that those with the highest contribution to this topic should be the 45 (Sq. root (2016) ≈ 44.89) authors with the highest number of papers. In the discrete count of authors, it was found that there were 73 authors with 4 or more papers and 40 authors with 5 or more papers, the latter being considered the prolific authors (Figure 3).

**Figure 4:**
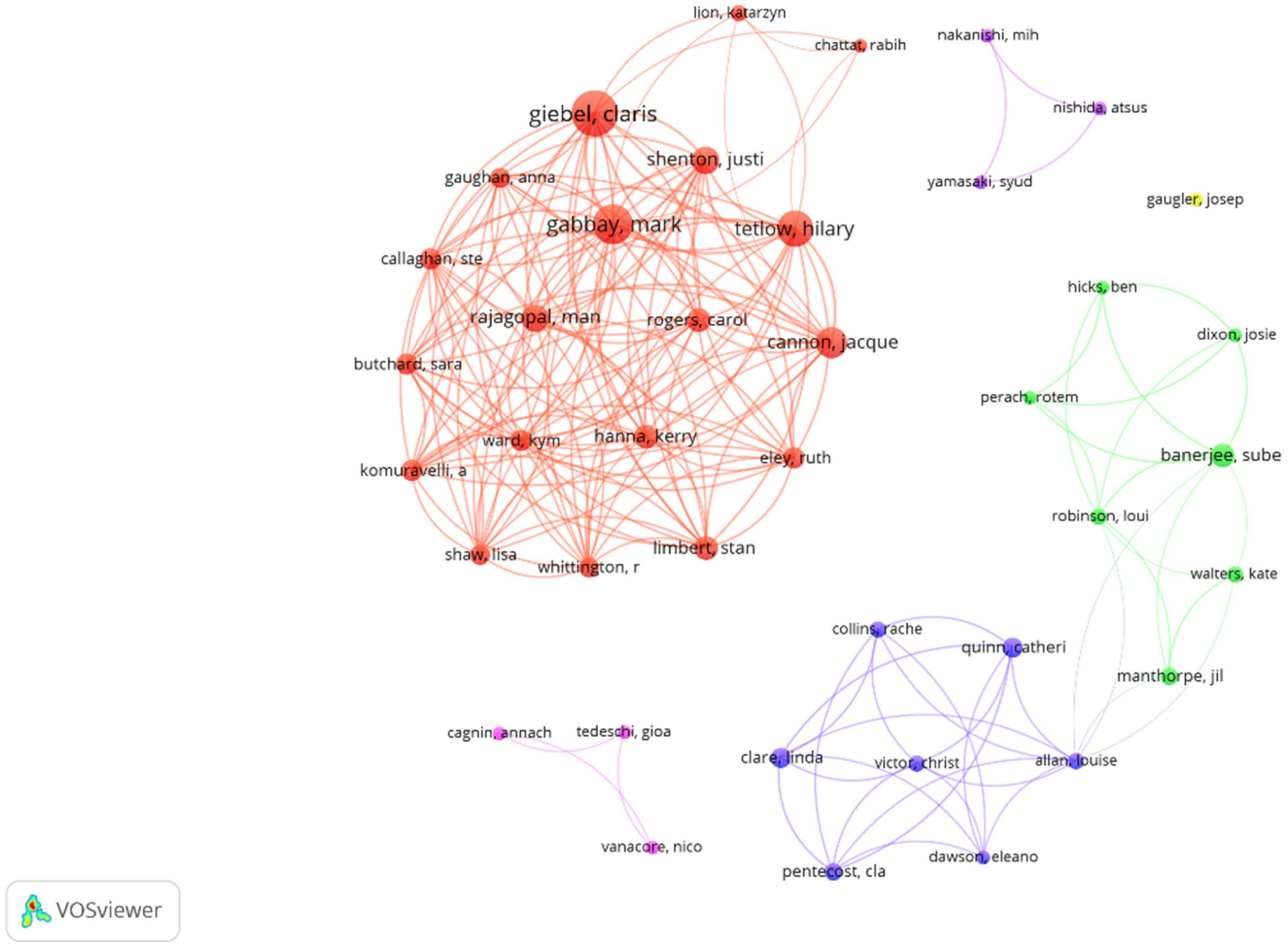
Prolific authors and collaborative networks. Each colour denotes a co-authorship cluster.

Figure 4 shows the 40 prolific authors and the collaborative network formed between them. The size of the nodes is based on the number of papers, and the colour represents the cluster to which they belong. There are five main collaborative networks among which the one formed by authors such as Claris Giebel, Mark Gabbay or Man Rajagopal (red cluster), and the collaborative network of Sube Banerjee, Josie Dixon or Jil Manthorpe (green cluster) connected to the network formed by Cla Pentecost, Louise Allan or Victor Christ (purple cluster).

**Figure 5:**
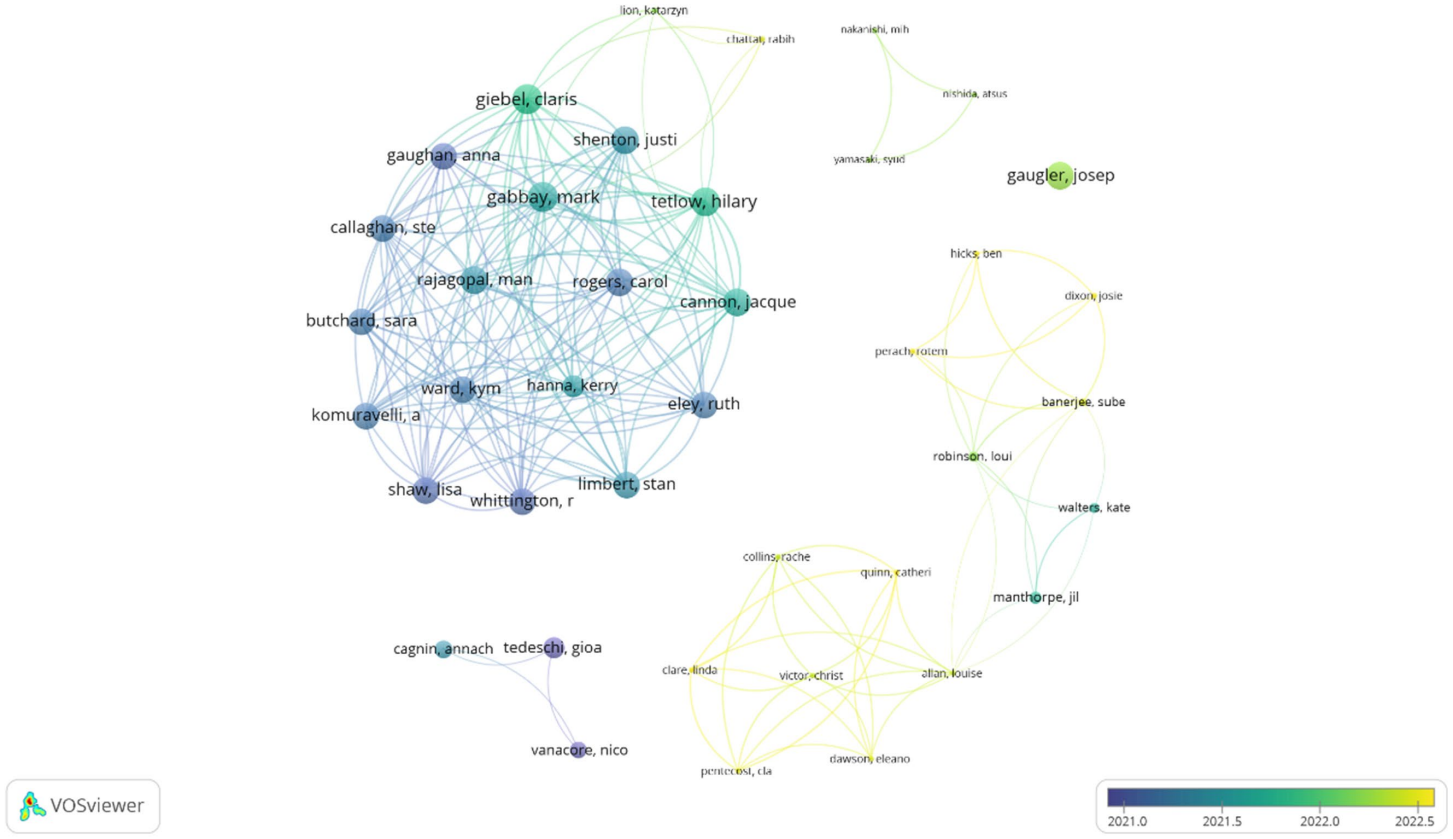
Prolific authors and collaborative networks based on the year of their publications.

Figure 5 shows these same prolific co-authors, although the visual scale is a function of the average year of publication. In this sense, the cluster formed by Cla Pentecost, Louise Allan or Victor Christ stands out, being the authors with the most recent average publication (purple cluster).

### 3.4 Cited papers

**Figure 6:**
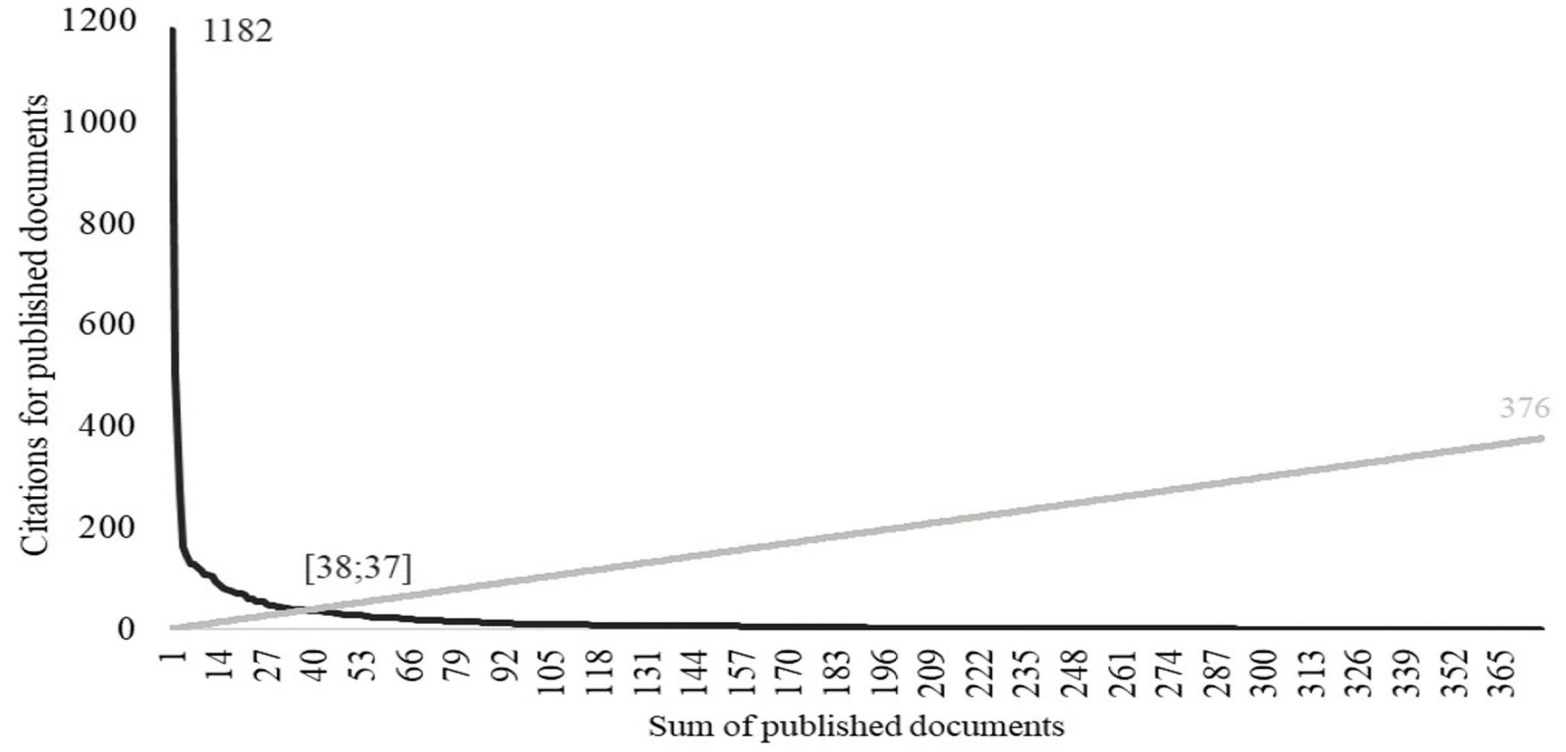
Number of documents by number of citations.

When analysing the citations of the 376 documents by applying the h-index, 37 documents with 38 or more citations stood out. Figure 6 shows the relationship between the number of documents with the highest to the lowest citations number, and the citations number, setting the cut-off point at 37 documents and 38 citations. These 37 documents were considered to be the most relevant to the topic (see Table 1).

**Table 1:** Web of Science categories with the greatest number of documents.

WoS categories	Docs. number	%^a^
Geriatrics Gerontology	129	34.3
Gerontology	121	32.2
Psychiatry	74	19.7
Neurosciences	41	10.9
Nursing	36	9.6
Public Environmental Occupational Health	34	9.0
Medicine General Internal	27	7.2
Clinical Neurology	25	6.7
Health Care Sciences Services	22	5.9

WoS, Web of Science; Docs. number, number of documents; %, percentage.

a Documents can be included in more than one category.

Table 2 shows the 37 most cited documents, their authors, titles, years of publication and number of citations in documents indexed in WoS. Among these, “2021 Alzheimer’s Disease Facts and Figures,” published under the authorship of Alzheimer’s Association thanks the authors Joseph Gaugler, PhD, Bryan James, Tricia Johnson, Jessica Reimer, and Jennifer Weuve for their production was the most cited. This paper has been cited 1,182 times, more than double the second most cited paper. We found 16 prominent authors among the prolific authors, all of them with 5 papers among the most cited, all belonging to the red cluster in Figure 4.

**Table 2:** Most cited papers.

Authors	Document	Journal	Times cited	WoS index
Alzheimer’s Association (34)	2021 Alzheimer’s disease facts and figures	Alzheimer’s & Dementia	1,182	SCI-E; SSCI
Alzheimer’s Association (35)	2022 Alzheimer’s disease facts and figures	Alzheimer’s & Dementia	504	SCI-E; SSCI
Brown et al. (36)	Anticipating and mitigating the impact of the COVID-19 pandemic on Alzheimer’s disease and related dementias	American Journal of Geriatric Psychiatry	273	SCI-E; SSCI
Giebel et al. (37)	Impact of COVID-19 related social support service closures on people with dementia and unpaid carers: a qualitative study	Aging & Mental Health	164	SCI-E; SSCI
Verbeek et al. (38)	Allowing visitors back in the nursing home during the COVID-19 crisis: a Dutch national study into first experiences and impact on well-being	Journal of the American Medical Directors Association	146	SCI-E; SSCI
Cohen et al. (39)	Living with dementia: increased level of caregiver stress in times of COVID-19	International Psychogeriatrics	129	SCI-E; SSCI
Altieri and Santangelo (40)	The psychological impact of COVID-19 pandemic and lockdown on caregivers of people with dementia	American Journal of Geriatric Psychiatry	128	SCI-E; SSCI
Cagnin et al. (18)	Behavioral and psychological effects of coronavirus disease-19 quarantine in patients with dementia	Frontiers in Psychiatry	123	SCI-E; SSCI
Lara et al. (41)	Neuropsychiatric symptoms and quality of life in Spanish patients with Alzheimer’s disease during the COVID-19 lockdown	European Journal of Neurology	114	SCI-E
Alzheimer’s Association (42)	2023 Alzheimer’s disease facts and figures	Alzheimer’s & Dementia	107	SCI-E
Cuffaro et al. (43)	Dementia care and COVID-19 pandemic: a necessary digital revolution	Neurological Sciences	107	SCI-E
Giebel et al. (44)	A UK survey of COVID-19 related social support closures and their effects on older people, people with dementia, and carers	International Journal of Geriatric Psychiatry	104	SCI-E; SSCI
Boutoleau-Bretonnière et al. (45)	The effects of confinement on neuropsychiatric symptoms in Alzheimer’s disease during the COVID-19 crisis	Journal of Alzheimer’s Disease	93	SCI-E; SSCI
Greenberg et al. (46)	Impact of COVID-19 pandemic restrictions on community-dwelling caregivers and persons with dementia	Psychological Trauma-Theory Research Practice and Policy	90	SSCI
Lai et al. (47)	The protective impact of telemedicine on persons with dementia and their caregivers during the COVID-19 pandemic	American Journal of Geriatric Psychiatry	80	SCI-E; SSCI
Savla et al. (48)	Dementia caregiving during the stay-at-home phase of the COVID-19 pandemic	Journals of Gerontology Series B-Psychological Sciences and Social Sciences	77	SCI-E; SSCI
Tsapanou et al. (49)	The impact of COVID-19 pandemic on people with mild cognitive impairment/dementia and on their caregivers	International Journal of Geriatric Psychiatry	76	SCI-E; SSCI
Rainero et al. (50)	The impact of COVID-19 quarantine on patients with dementia and family caregivers: a nation-wide survey	Frontiers in Aging Neuroscience	73	SCI-E; SSCI
Simonetti et al. (51)	Neuropsychiatric symptoms in elderly with dementia during COVID-19 pandemic: definition, treatment, and future directions	Frontiers in Psychiatry	71	SCI-E; SSCI
Budnick et al. (52)	Informal caregivers during the COVID-19 pandemic perceive additional burden: findings from an *ad-hoc* survey in Germany	BMC Health Services Research	70	SCI-E; SSCI
Vaitheswaran et al. (53)	Experiences and needs of caregivers of persons with dementia in India during the COVID-19 pandemic-a qualitative	American Journal of Geriatric Psychiatry	68	SCI-E; SSCI
Geddes et al. (54)	Remote cognitive and behavioral assessment: report of the Alzheimer Society of Canada Task Force on dementia care best practices for COVID-19	Alzheimer’s & Dementia: Diagnosis, Assessment & Disease Monitoring	60	ESCI
Borges-Machado et al. (55)	The effects of COVID-19 home confinement in dementia care: physical and cognitive decline, severe neuropsychiatric symptoms and increased caregiving burden	American Journal of Alzheimer’s Disease and other Dementias	59	SCI-E; SSCI
Barguilla et al. (56)	Effects of COVID-19 pandemic confinement in patients with cognitive impairment	Frontiers in Neurology	55	SCI-E
Giebel et al. (57)	COVID-19-related social support service closures and mental well-being in older adults and those affected by dementia: a UK longitudinal survey	BMJ Open	54	SCI-E; SSCI
Giebel et al. (58)	Decision-making for receiving paid home care for dementia in the time of COVID-19: a qualitative study	BMC Geriatrics	54	SCI-E; SSCI
Cohen et al. (59)	COVID-19 epidemic in Argentina: worsening of behavioral symptoms in elderly subjects with dementia living in the community	Frontiers in Psychiatry	48	SCI-E; SSCI
Tuijt et al. (60)	Life under lockdown and social restrictions—the experiences of people living with dementia and their carers during the COVID-19 pandemic in England	BMC Geriatrics	46	SCI-E; SSCI
Hanna et al. (61)	Emotional and mental wellbeing following COVID-19 public health measures on people living with dementia and carers	Journal of Geriatric Psychiatry and Neurology	46	SCI-E; SSCI
Carpinelli Mazzi et al. (62)	Time of isolation, education and gender influence the psychological outcome during COVID-19 lockdown in caregivers of patients with dementia	European Geriatric Medicine	45	SCI-E; SSCI
Liu et al. (63)	Dementia wellbeing and COVID-19: review and expert consensus on current research and knowledge gaps	International Journal of Geriatric Psychiatry	43	SCI-E; SSCI
Baschi et al. (64)	Changes in motor, cognitive, and behavioral symptoms in Parkinson’s disease and mild cognitive impairment during the COVID-19 Lockdown	Frontiers in Psychiatry	42	SCI-E; SSCI
Leontjevas et al. (65)	Challenging behavior of nursing home residents during COVID-19 measures in the Netherlands	Aging & Mental Health	41	SCI-E; SSCI
Iaboni et al. (66)	Achieving safe, effective, and compassionate quarantine or isolation of older adults with dementia in nursing homes	American Journal of Geriatric Psychiatry	40	SCI-E; SSCI
Paananen et al. (67)	The impact of COVID-19-related distancing on the well-being of nursing home residents and their family members: a qualitative study	International Journal of Nursing Studies Advances	39	ESCI
Azevedo et al. (68)	Impact of social isolation on people with dementia and their family caregivers	Journal of Alzheimer’s Disease	39	SCI-E; SSCI
Van Maurik et al. (69)	Psychosocial effects of corona measures on patients with dementia, mild cognitive impairment and subjective cognitive decline	Frontiers in Psychiatry	39	SCI-E; SSCI

WoS, Web of Science; SCI-Expanded, Science Citation Index Expanded; SSCI, Social Sciences Citation Index.

### 3.5 Region/country of collaboration

**Figure 7:**
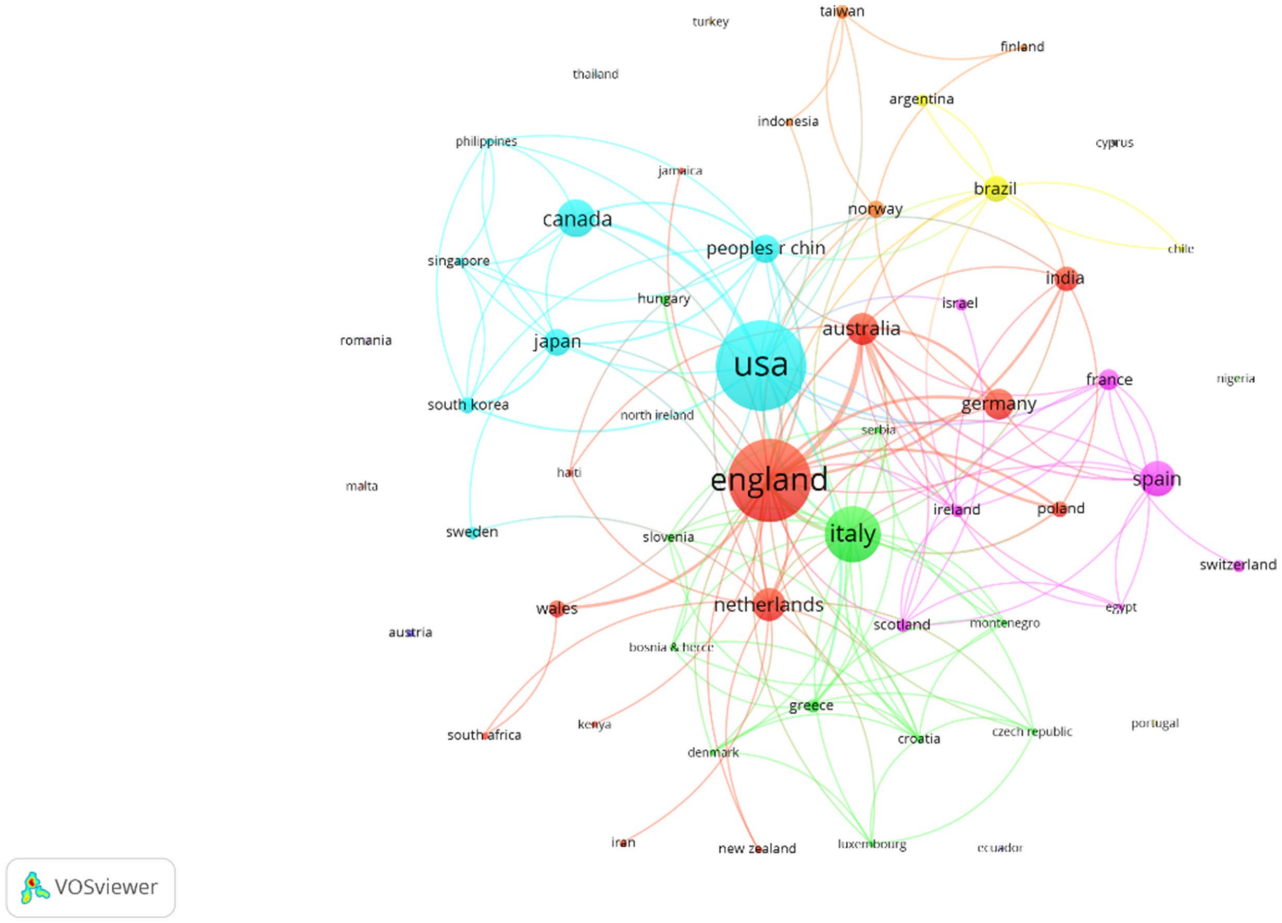
Countries with the highest number of papers and their geographical collaborations. Colours represent the geographical co-authorships.

Figure 7 presents the collaborations between the countries involved in the generation of information on dementia care research in COVID-19. The USA (96 papers, 9.2 citations per article), England (84 papers, 11.9 citations per article), Italy (44 papers, 20.6 citations per article), Canada (24 papers, 20.8 citations per article) and Spain (21 papers, 15.6 citations per article) with the highest number of papers/citations, stood out. Six collaboration clusters were found, highlighting four large clusters led by England (red cluster, 13 countries/regions), Italy (green cluster, 11 countries/regions), the USA (light blue cluster, 8 countries/regions) and Spain (pink cluster, 7 countries).

### 3.6 Journals

A total of 158 journals were found and grouped into three terciles after applying Bradford’s law (Table 3): Core (9 journals, 129 documents); Zone I (41 journals, 139 documents); and Zone II (158 journals, 158 documents).

**Table 3:** Bradford’s zones.

Zone	Documents number (%)		Journals (%)		Bradford multipliers	Journals (theoretical series)	
CORE	129	34%	9	6%		9 × (*n*0)	9
Zone I	139	37%	41	26%	4.6	9 × (*n*1)	32
Zone II	108	29%	108	68%	2.6	9 × (*n*2)	117
Total	376	100%	158	100	3.6		153
						% Error	3.2%

Table 4 shows the nine journals that made up the publication core, indicating the name of the journal, the number of documents published in each journal, the accumulated citations, the thematic category in which they publish, and in which index they were found to be indexed.

**Table 4:** Magazines that make up the core zone.

Journal	Docs. number	Citation number	WOS category	Quartile	WoS index
Journal of Alzheimer’s Disease	21	400	Neurosciences	Q2	SCI-E
Frontiers in Psychiatry	19	526	Psychiatry	Q2	SCI-E; SSCI
BMC Geriatrics	18	187	Gerontology	Q1	SCI-E; SSCI
Dementia-International Journal of Social Research and Practice	17	73	Gerontology	Q3	SCI-E
International Journal of Geriatric Psychiatry	15	274	Gerontology	Q2	SCI-E; SSCI
International Journal of Environmental Research and Public Health	13	111	Environmental Sciences	Q2 (2021)	SCI-E; SSCI
Aging & Mental Health	10	240	Gerontology	Q2	SCI-E; SSCI
Clinical Gerontologist	8	45	Gerontology	Q2	SCI-E; SSCI
Gerontology and Geriatric Medicine	8	48	Geriatrics & Gerontology	Q3	SCI-E

Docs. number, number of documents; WoS, Web of Science; SCI-Expanded, Science Citation Index Expanded; SSCI, Social Sciences Citation Index.

### 3.7 Author keywords

**Figure 8:**
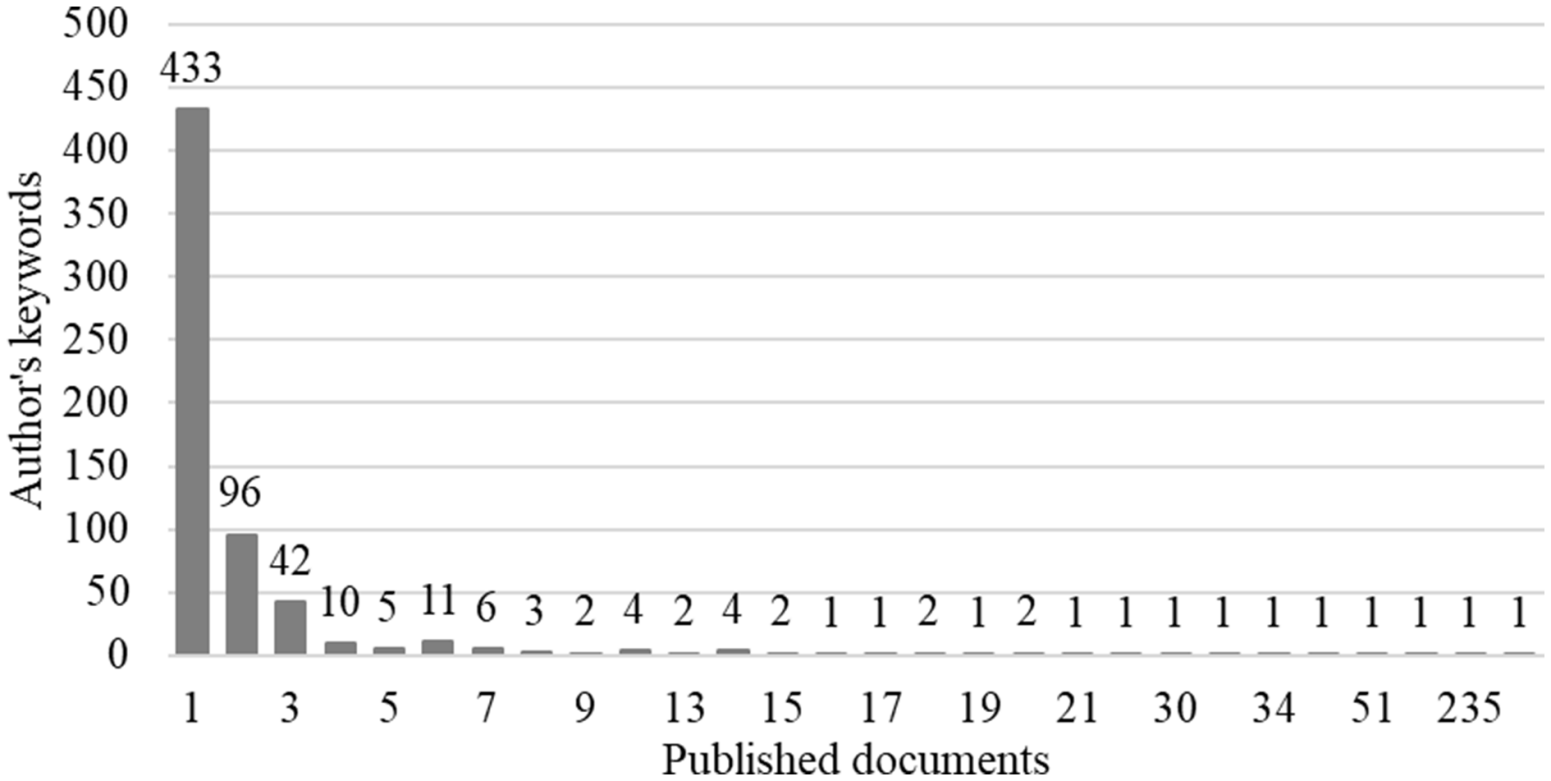
Relationship between the keywords and the number of documents in which they appeared.

After keywords normalisation, a total of 637 author keywords were found. Applying Zipf’s law it was estimated that the keywords of most interest to authors should be the 25 (square root of 637) with the most occurrences in the set of articles. In the discrete keyword and article counts, 25 keywords with 23 or more occurrences were found, taking these as the keywords of most interest to authors (Figure 8).

**Figure 9:**
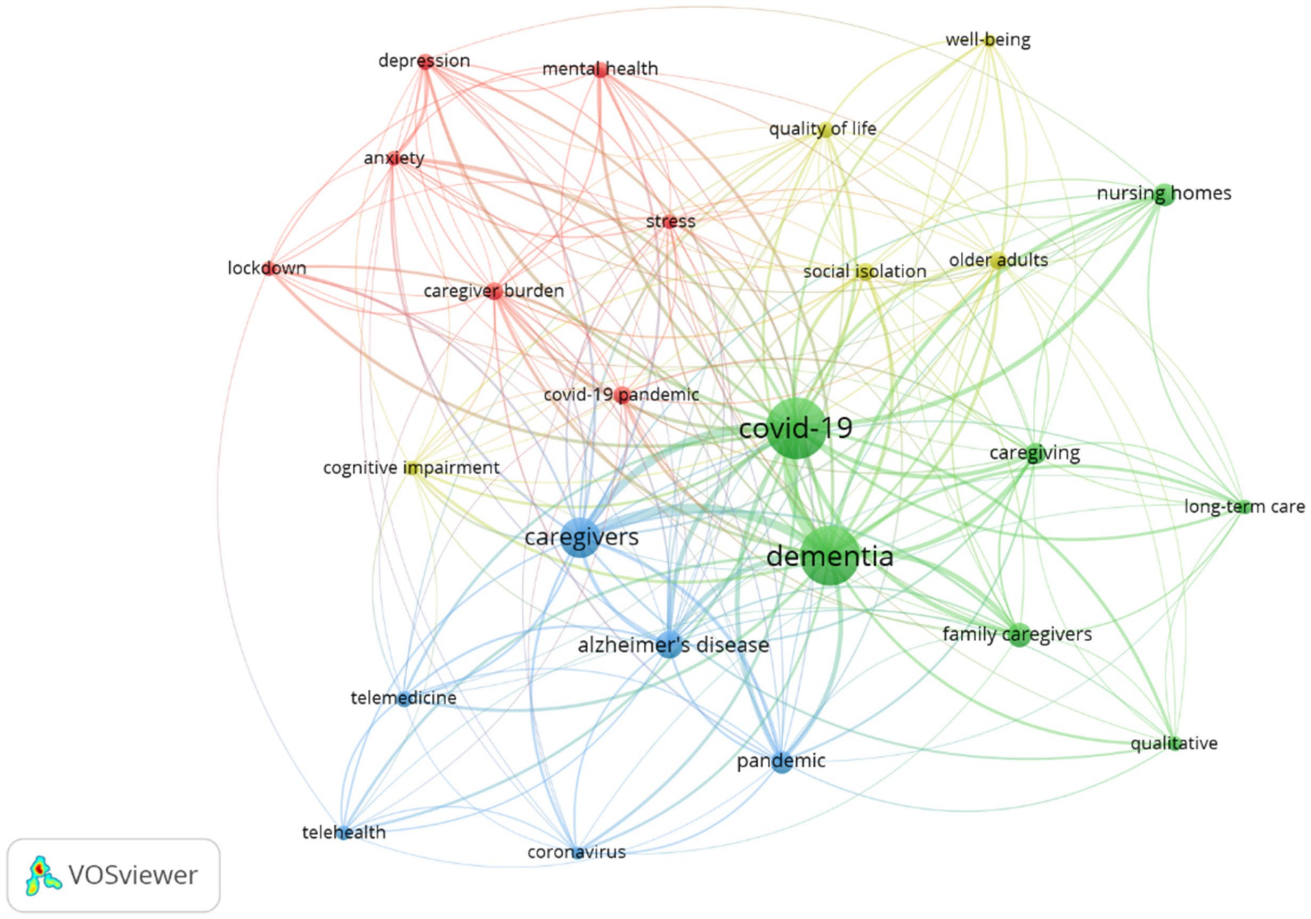
Clusters according to keywords. Each colour represents a cluster.

The 25 keywords most relevant to the authors formed four clusters (Figure 9). The red cluster is more related to stress, anxiety, depression, mental health and burden of care; the blue cluster is more linked to telemedicine and telehealth concerning the pandemic; the green cluster refers to family caregivers, long-term care, and nursing homes; and the yellow cluster focuses on well-being variables, quality of life, cognitive impairment, social isolation and older adults.

## 4 Discussion

This study represents the first bibliometric analysis that has undertaken a scientific mapping of the state of publications related to the care of people with dementia in the context of COVID-19. This manuscript provides a quantitative and systematic analysis of the dynamic evolution of this topic of study, including all articles in WoS-indexed journals regarding the care of dementia from 2020 to 2023. A total of 370 documents met the inclusion and exclusion criteria and were selected for further analysis providing information to researchers on the current status of publications on this topic in the matters of most prevailing study trends, referential documents, most prominent co-authors, and journals with the greatest interest for this subject.

During the period under analysis, the number of publications increased from 2020, the year in which COVID-19 became a globally relevant issue, to the two following years 2021 and even more in 2022. A previous bibliometric analysis has shown that the number of documents regarding dementia family caregivers experienced a growth face since 1998 to 2018 (70). After an initial phase of stability since 1988, when the first item on the topic was published (70). Other research that focused on Montessori interventions in patients with dementia reported a gradual increase in the number of publications on the topic, from the year 2000 until 2020 when the number of research items increased significantly (71). According to the available evidence, research on people with dementia caregivers has grown in interest since the year 2000 and even more in the last decade, according to the number of publications on the subject. Unfortunately, no other bibliometric study has analyzed the evolution of publications on the subject during the years 2021 and 2022.

The top three WoS categories with the highest number of publications in the period under study were “Geriatrics Gerontology,” “Gerontology” and “Psychiatry.” The first two with more than 100 articles published during this period. In terms of journals, a bibliometric study focused on health and interventions in caregivers of people with dementia, found that between the years 1988 and 2018, the three journals with the highest number of published papers were The International Journal of Geriatric Psychiatry, International Psychogeriatrics, and Aging & Mental Health (70). Another study focused on informal caregivers found that the top three most prolific journals on this topic until 2023 were BMC Geriatrics, Aging & Mental Health, and The International Journal of Environmental Research and Public Health (72). In the present study, it was found that since 2020, the three journals with the highest number of publications on the subject were the Journal of Alzheimer’s Disease, Frontiers in Psychiatry, and BMC Geriatrics. The previously mentioned journals International Journal of Geriatric Psychiatry, Aging & Mental Health, and The International Journal of Environmental Research and Public Health were also identified as part of the nine core journals on the topic during the COVID-19 period.

Co-authors with 5 or more publications were identified as prolific. Thus, Clarissa Giebel, Mark Gabbay and Hilary Tetlow, all of them affiliated with the University of Liverpool, were the top three authors with the highest number of publications, with 23, 19 and 17 documents respectively, during the studied period. It should also be noted that between them they formed a collaborative network so that many of their articles were co-authored by at least two of them. In other research, Shi et al. (70) only considered the number of documents published by the first author. This pointed out Mary S. Mittelman from New York University, Henry Brodaty from the University of New South Wales, and Joseph E. Gaugler from the University of Minnesota as the three most prolific authors since the beginning of the research on caregivers of people with dementia until 2018 (70). Sadly, the collaborative relationships between researchers were not explored in this study.

Among the most cited articles, the two publications with the largest number of citations are authorized by the Alzheimer’s Association, both of them focused on describing the impact of this disease on caregivers and society (34, 35). The third leading manuscript in terms of citations cited more than 200 times, focuses on the risks of COVID-19 on people with Alzheimer’s disease and related dementias and proposes strategies to manage and minimize the negative impact of COVID-19 on this population (36). It is worth noticing that none of these three articles was published in any of the core journals on the topic.

Regarding the countries o territories with greater implications in the topic, the USA, England, Italy, Canada, and Spain stand out in terms of publications and citations. Each one of them leads a cluster of collaborations among researchers from different nations, except for Canada which is part of the cluster led by the USA. The three most prolific co-authors of the subject were all affiliated with an English university. In another bibliometric analysis regarding informal caregivers, these same countries were reported to be relevant in the subject, but China, Germany and Australia also appeared as important nations in the research on this topic (72).

Finally, the analysis of the keywords allowed us to see the existence of four clusters of words, with the themes of mental health, caregivers, well-being/quality of life and telemedicine. This indicates that the focus of much of the research on this topic during the COVID-19 period has been on the mental health, quality of life and well-being of people with dementia caregivers, including both family and professional caregivers (10, 11).

However, other bibliometric analyses focused on family caregivers analyzed research from 1988 to 2018, and up to 5 clusters were reported (70). These clusters did not have easily identifiable themes, but among them, the following could be identified: Alzheimer’s disease, family caregivers, burden, mental health and symptoms (70). In another study focused on informal caregiving, the relationships between keywords were not studied but the most used terms were, finding that dementia, family caregivers, quality of life and health, were the most used words, alongside other terms such as management, family and stress, that were used less frequently (72). The main difference between the present article and other similar bibliometric research is the existence of keywords related to telemedicine, a direct consequence of the fact that our research includes the years from 2020 onwards, and telemedicine and telehealth were of great importance during these COVID-19 years.

The practical implications of this study regarding research in dementia care in the context of COVID-19 are significant, particularly in light of the pandemic’s impact on public health and its disproportionate effects on individuals with dementia disease and caregivers. By analyzing bibliometric data from the WoS Collection, this study identifies key trends and patterns including the top authors, institutions, countries, and journals contributing to the field. A key finding is the volume of annual publications related to COVID-19 and dementia patient care in just 3 years, highlighting the existence of a substantial body of research on this important topic. By identifying the most productive journals, researchers can better understand the publication landscape and choose appropriate venues for disseminating their work. Furthermore, analyzing the most frequently cited articles provides valuable insights into the research themes and questions driving the field’s progress. In terms of collaboration, this study reveals essential networks and connections between researchers, institutions, and countries working on dementia care in the context of COVID-19. By mapping these collaborations, researchers can identify potential partners and build stronger networks to advance knowledge in this area. Moreover, the identification of research hotspots and emerging trends helps researchers stay informed about the latest developments and adjust their research agendas accordingly.

In this line, based on the results obtained and as future lines of research it could be interesting to address studies that evaluate in depth the use of telemedicine in dementia care during COVID-19, analyzing its effectiveness, challenges and potential for long-term integration. Similarly, research could investigate the specific mental health challenges faced by caregivers of people with dementia during the pandemic and whether they persist, and consequently develop specific interventions and support mechanisms. Finally, one of the most interesting future lines of research would be to investigate the long-term consequences of COVID-19 on dementia care practices and patient outcomes and thus assess the need for adjustments and adaptations.

The main limitation of this study is that it is based exclusively on data from the WoS Core Collection, which may introduce biases due to the exclusion of relevant literature from other databases. This is quite common within bibliometric studies due to the incompatibility of the different sets of databases (73) in comparative terms, mainly in the comparison by impact due to their different coverage of journals, proceedings and books, which forced us to limit ourselves to a specific set of databases (in this case WoS), to perform an analysis on a broader coverage of data fields and metadata. In addition, this study has focused on the care of people with dementia in the context of COVID-19 from a generic point of view, another future line of research on the bibliometric analysis of this topic, it would be interesting to specifically address different types of caregivers or contexts of care (e.g., formal or informal), including the analysis of more specialized documents, increasing the amount of relevant information on the corresponding topic. Despite this limitation, this study provides valuable information on global trends in dementia care research in the context of COVID-19, highlighting the importance of continued research in this critical area.

## 5 Conclusion

This is the first bibliometric analysis conducted on research on the caregiving of people with dementia in the context of COVID-19. This bibliometric analysis provides an overview of the research on this subject, helping researchers to have a macro-perspective of how the topic is structured and how it has evolved since 2020. The number of publications on this theme increased from the year 2020 to 2021 and 2022. Two journals, Geriatrics Gerontology and Gerontology, published more than 100 documents on the topic in this period. A total of 9 journals were considered core journals. The top three most prominent authors were all affiliated with the University of Liverpool and formed a collaborative network with multiple publications co-authorized by them. The USA, England, Italy, Canada, and Spain were the countries that stood out in terms of the number of publications. The collaborations among these nations were also described. Mental health, caregivers, well-being/quality of life and telemedicine, were the main themes of the four clusters formed by the keywords in the research topic.

## Data availability statement

The original contributions presented in the study are included in the article/Supplementary material, further inquiries can be directed to the corresponding author.

## Author contributions

RG-G: Methodology, Writing – original draft. ÁD-Z: Formal analysis, Methodology, Writing – original draft. MM-M: Conceptualization, Writing – review & editing. DP-P: Formal analysis, Writing – review & editing. SB-F: Conceptualization, Writing – review & editing. LM-B: Conceptualization, Project administration, Writing – original draft.
